# Predictors of surgical management and its impact on outcomes for combined C1–C2 fractures: National registry study

**DOI:** 10.1051/sicotj/2025058

**Published:** 2026-01-06

**Authors:** Kristin Salottolo, W. Tyler Crawley, Kaysie Banton, David Acuna, Carlos H. Palacio, Darryl Auston, Peter Syre, David Bar-Or

**Affiliations:** 1 Swedish Medical Center, Trauma Research Department 601 E Hampden Avenue, Suite 100 Englewood CO 80113 USA; 2 Trauma Services Department, Swedish Medical Center 501 E. Hampden Ave Englewood CO 80113 USA; 3 Trauma Services Department, Wesley Medical Center 550 N Hillside Street Wichita KS 67214 USA; 4 Trauma Services Department, South Texas Health System McAllen 301 W Expy 83 McAllen TX 78503 USA; 5 Trauma Services Department, Lutheran Hospital 12911 W. 40th Ave Wheat Ridge CO 80401 USA; 6 Neurosurgery Department, Swedish Medical Center 500 E. Hampden Ave, Suite 200 Englewood CO 80113 USA

**Keywords:** Cervical spine fracture, Trauma, Mortality, Spine surgery

## Abstract

*Introduction*: Combined C1–C2 fractures are common upper cervical injuries with high morbidity and mortality. Controversy exists regarding which patients benefit from surgery because this is an understudied population with only class III evidence available. We examined surgical intervention and its impact on outcomes in patients with C1–C2 fractures. *Methods*: This retrospective cohort study of the National Trauma Data Bank included patients admitted between 1/2017 and 1/2023 for combined C1–C2 fractures (ICD-10 diagnosis codes S12.0 and S12.1). Exclusions were admission to a level III-V or non-trauma center, not admitted (died or discharged from the ED), and non-index/readmission. The first aim was to identify predictors of surgical intervention (vertebral fusion or internal fixation); multivariate backward regression included the following covariates: Patient demographics, injury severity, concomitant injuries, and specific C1 and C2 fractures. The second aim was to compare hospital outcomes between operative and nonoperative groups utilizing a propensity-matched (1:1) analysis: Mortality, ICU admission, complications, and hospital and ICU LOS. *Results*: There were 19,264 patients, and 3,759 (19.5%) were surgically managed. The adjusted odds of surgical intervention were greater with unstable injuries (displaced C1 fracture, displaced C2 fracture, spinal cord injury, vertebral ligament dislocation), specific C1 and C2 fractures (odontoid fracture, Jefferson burst fracture, posterior arch fracture), whereas surgical intervention odds decreased for frailty (mFI ≥2), ED hemodynamic instability, ED Glasgow coma score ≤8, and increasing age quintile. Propensity matching resulted in 6,710 well-matched patients. After matching, surgical intervention was associated with lower mortality (4.8% vs. 11.3%, *p* < 0.001) but higher ICU rates, longer LOS, and greater complication rates compared to the nonoperative group. *Conclusion*: This study of nearly 20,000 patients with combined C1–C2 fractures provides class II evidence for surgical intervention, highlighting the balance between injury characteristics and patient resilience. Surgical intervention was associated with a significant survival benefit, emphasizing its role in select patients.

## Introduction

The unique anatomy and biomechanical relationship between the first and second cervical vertebrae (C1 and C2), also known as the atlas and axis, result in a wide array of injury patterns [1]. Combined atlas and axis fractures are common [2]. Approximately 40% of atlas fractures have a concomitant axis fracture [3]; this rate increases to 57% in geriatric patients [4]. Combined fractures have higher morbidity and greater biomechanical instability than isolated fractures [5].

Although combined C1–C2 injuries are not infrequent, there are few published studies in this population; the largest cohort was 107 patients [4] and the largest systematic review included 230 patients [6]. Expert-based consensus recommendations for surgical management of combined C1–C2 fractures by the AANS/CNS were based on class III evidence only [7]. Thus, the optimal management of upper cervical fractures is controversial, as they can be managed operatively or nonoperatively with no clear criteria for surgical intervention.

Several authors have proposed surgical management of combined fractures based on the C2 fracture type and location, [7–10] as well as the presence of ligamentous injuries [6, 9]. The frequency of upper cervical injuries among the elderly is a major consideration for management due to patient comorbidity and frailty, and the aging pathology resulting in weaker blood supply and bone quality [11, 12]. These factors may lend greater risk to the patient than the fracture pattern or its subsequent management [13–15].

Because this is an understudied population, we sought to use a large national registry to examine patients with combined C1–C2 fractures. The primary aim was to identify covariates that increased the likelihood of surgical intervention. The secondary aim was to determine the effect of surgical management on in-hospital outcomes.

## Materials and methods

This study utilized the National Trauma Databank (NTDB), which is the largest aggregation of admissions due to traumatic injury in the United States. A trauma patient is defined as sustaining a traumatic injury with International Classification of Diseases (ICD)-10 injury codes S00-S99, T07, or T14. Study specific inclusion criteria were index admission from January 1, 2017 to January 1, 2023 and diagnoses of both C1 and C2 fractures using ICD-10 codes beginning with S12.0 and S12.1 Exclusions were as follows: Admission to a lower level (≥III) or non-trauma center, readmissions, and patients not admitted (ED discharge home, jail, against medical advice, morgue, and transfer to another facility).

The NTDB uses a standardized data dictionary and quality checks [16]. Covariates included [17] injury characteristics (cause of injury, injury severity score (ISS; <25 vs. ≥25 indicating polytrauma), ED Glasgow Coma Scale (GCS) of severe (3–8) vs. mild/moderate (9–15), ED hemodynamic instability (systolic blood pressure <90 mmHg or pulse >120 beats/minute); frailty (5-item modified frailty index (mFI-5), <2 vs. ≥2), [17]; concomitant injuries using ICD-10 diagnosis codes (spinal cord injury, traumatic brain injury, cervical ligamentous sprain, vertebral ligament dislocation); specific C1 fracture patterns (Posterior arch, Jefferson burst fracture, lateral mass fracture, other C1 fractures); and specific C2 fractures patterns (odontoid type II, odontoid type I/III, non-odontoid fractures of Hangman’s and base fractures); fracture displacement; and demographics (age quintile, sex, race). Exact ages of individuals older than 89 are considered protected health information (PHI) and were recorded as 90 or above. Patients >89 years were denoted as having an age of 90 to define age quartiles.

### Statistical analysis

Analyses were performed with SAS version 9.4 (SAS Institute, Cary, NC). Frequencies and descriptive statistics were used to characterize the study population using Pearson chi-square tests. An alpha <0.05 was used for statistical significance.

Surgical intervention was defined by ICD-10 procedure codes for vertebral fusion or internal fixation. A multivariate logistic regression analysis was used to examine covariates associated with surgical intervention. There were no formal hypotheses tested; following iterative backwards selection of covariates from the model based on *p* > 0.05, all remaining covariates independently associated with surgery were reported. The model was evaluated for collinearity using a correlation coefficient >0.50 and the Akaike information criterion. Age and cause of injury were highly collinear. Additionally, there was collinearity with odontoid type II fractures and non-odontoid fractures. Cause of injury and non-odontoid fracture were not included as covariates in the regression model.

The secondary aim examined the following in-hospital outcomes: Mortality, ICU admission, development of a complication, nonsurvival (discharge destination to hospice or morgue), and hospital and ICU LOS. Evaluation of our secondary aim was performed using a propensity-matched analysis with a 1:1 ratio and caliper distance of 0.005. After matching, McNemars tests analyzed the difference in categorical outcomes (in-hospital mortality, ICU admission, complication, nonsurvival) and Wilcoxon signed-rank tests analyzed differences in median hospital and ICU LOS.

## Results

### Population

**Figure 1:**
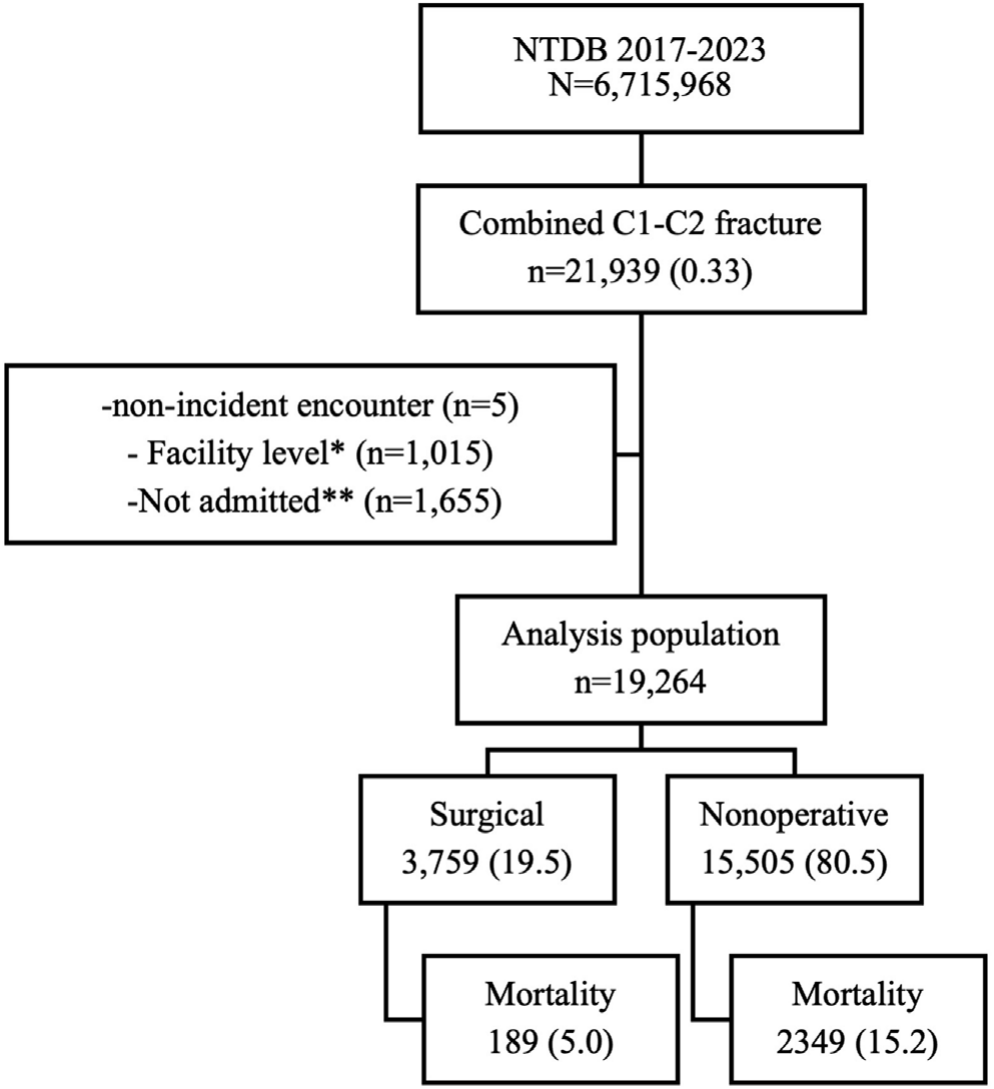
Study population. *Lower level (III–V) or non-trauma center designation/accreditation status. **ED discharge disposition to home, jail, transfer, against medical advice or morgue.

The records of 19,247 patients with combined C1–C2 fractures were identified for analysis (Figure 1). The median age was 76 years (interquartile range: 62–86), and 38% of patients were considered frail based on a modified frailty index ≥2. There were similar proportions of men and women (51:49). The majority (69%) were injured in a fall, with 22% injured in a motor vehicle crash (MVC), 5% injured in an auto-pedestrian or auto-bike crash, and 4% by another cause. Most patients had displaced upper cervical fractures (C1, 68%; C2, 78%). The most common C2 fracture was an odontoid type II fracture (48%), and the most common C1 fracture was a posterior arch fracture (29%). Most patients did not have severe injuries, based on ISS ≥25 (11%), ED GCS 3–8 (12%), and ED hemodynamic instability (8%). Concomitant injuries were also infrequent: 18% had TBI, 10% had SCI (78% were partial cord injuries), 9% had cervical ligament sprain, and 6% had vertebral ligament dislocation.

### Surgical management

Nearly twenty percent (*n* = 3,758) of patients were surgically managed. As shown in Table 1, there were significant differences between surgical and nonsurgical groups for almost every covariate. Surgically managed patients were less likely to be frail and to have severe injuries (ISS ≥25, GCS ≤8, hemodynamic instability) and were more likely to be younger, male, have concomitant injuries, odontoid fractures, and displaced C1/C2 fractures.

**Table 1:** Univariate associations with surgical intervention.

Characteristic, *n* (%)	Nonsurgical (*n* = 15,505)	Surgical (*n* = 3,759)	*p*-Value
Age quintile			**<0.001**
<55	2506 (16.2)	817 (21.7)	
55–69	2705 (17.5)	1009 (26.8)	
70–79	3165 (20.4)	1019 (24.4)	
80–86	3138 (20.2)	610 (16.2)	
≥87	3991 (25.7)	304 (8.1)	
Frail (mFI-5 ≥2)	6069 (39.1)	1202 (32.0)	**<0.001**
Male sex	7597 (49.1)	2241 (59.9)	**<0.001**
White race	13410 (86.5)	3189 (84.8)	**0.009**
Cause of injury			**<0.001**
Fall	10929 (70.5)	2387 (63.5)	
Vehicle occupant	3293 (21.2)	1009 (26.8)	
Bicycle or pedestrian	675 (4.4)	227 (6.0)	
All other causes	608 (3.9)	136 (3.6)	
ED hemodynamic instability	1325 (8.6)	223 (5.9)	**<0.001**
ED GCS 3–8 (severe)	1894 (12.8)	285 (7.9)	**<0.001**
ISS ≥25 (polytrauma)	1720 (11.1)	430 (11.5)	0.54
Concomitant injuries			
Cervical ligament sprain	1078 (7.0)	683 (18.2)	**<0.001**
Vertebral dislocation	687 (4.4)	463 (12.3)	**<0.001**
Traumatic brain injury (TBI)	2918 (18.8)	623 (16.6)	**0.001**
Spinal cord injury (SCI)	1281 (8.3)	739 (19.7)	**<0.001**
Fracture patterns			
Odontoid type II	7220 (46.6)	2031 (54.0)	**<0.001**
Odontoid type I/III	2358 (15.2)	663 (17.6)	**<0.001**
Non-odontoid C2 fracture	4445 (28.7)	957 (25.5)	**<0.001**
Jefferson burst	1080 (7.0)	397 (10.6)	**<0.001**
Posterior arch	4537 (29.3)	1114 (29.6)	0.65
Lateral mass	1978 (12.8)	429 (11.4)	**0.03**
Other C1 fracture	5027 (32.4)	1180 (31.4)	0.23
Displaced C2 fracture	11701 (75.5)	3329 (88.6)	**<0.001**
Displaced C1 fracture	10382 (67.0)	2754 (73.3)	**<0.001**

Bolding denotes statistical significance.

After adjustment, covariates that remained significantly associated with greater odds of surgical intervention were as follows: Displaced C1 fracture, displaced C2 fracture, spinal cord injury, vertebral ligament dislocation, and specific fracture patterns of odontoid type II, odontoid type I/III, Jefferson burst fracture, and posterior arch fracture (Table 2). At the same time, odds of surgical intervention decreased for ED GCS ≤8, hemodynamic instability in the ED, mFI ≥2, and increasing age quintile (Table 2).

**Table 2:** Stepwise multivariate regression modelling surgical intervention.

Covariate	OR (95% CI)	*p*-Value
Spinal cord injury	2.39 (2.13–2.69)	**<0.001**
Vertebral ligament dislocation	2.16 (1.86–2.51)	**<0.001**
Displaced C2 fracture	2.15 (1.90–2.43)	**<0.001**
Cervical ligament sprain	2.03 (1.81–2.28)	**<0.001**
Odontoid type II fracture	1.90 (1.74–2.08)	**<0.001**
Jefferson burst fracture	1.71 (1.46–1.99)	**<0.001**
Male (vs. female)	1.24 (1.14–1.34)	**<0.001**
Odontoid I/III fracture	1.22 (1.08–1.38)	**0.001**
Displaced C1 fracture	1.22 (1.09–1.35)	**<0.001**
Posterior arch fracture	1.12 (1.02–1.22)	**0.01**
Frailty ≥2 (MFI-5)	0.91 (0.83–0.99)	**0.04**
ED hemodynamic instability	0.72 (0.61–0.85)	**<0.001**
ED GCS ≤8	0.31 (0.27–0.37)	**<0.001**
Age (ref: Age <55)	55–69: 0.96 (0.85–1.09)	0.54
	70–79: 0.75 (0.66–0.85)	**<0.001**
	80–86: 0.44 (0.38–0.50)	**<0.001**
	≥87: 0.17 (0.14–0.20)	**<0.001**

Bolding denotes statistical significance. Model c-statistic: 0.75. Variables removed from the model with backward elimination: Race (*p* = 0.89), lateral mass fracture (*p* = 0.28), TBI (*p* = 0.24), Other C1 fracture (*p* = 0.11), and ISS ≥ 25 (*p* = 0.09).

### Outcomes

In-hospital mortality was 13.2%; further, one in six (16.6%) patients either died in hospital or were discharged to hospice care (nonsurvival). There was substantial morbidity in this population: 55.8% were admitted to the ICU, 16.1% developed a complication, and the median (IQR) hospital LOS and ICU LOS were 6 (4–10) days and 4 (2–7) days, respectively.

Before matching, operative intervention was associated with a significant survival benefit, both in-hospital and when discharge to hospice was considered (Table 3). However, patients who were operatively managed had significantly worse morbidity, including higher ICU admission, longer ICU LOS and hospital LOS, and a greater likelihood of developing a complication (Table 3).

**Table 3:** Outcomes of combined C1–C2 fractures, prior to and after propensity matching.

	All patients			Propensity matched		
Outcome, *n* (%) or median (IQR)	Nonoperative (15,505)	Operative (3,759)	*p*-value	Nonoperative (3,355)	Operative (3,355)	*p*-value
Mortality	2349 (15.2)	189 (5.0)	**<0.001**	380 (11.3)	161 (4.8)	**<0.001**
Morgue/hospice	2950 (19.0)	246 (6.5)	**<0.001**	481 (14.3)	214 (6.4)	**<0.001**
ICU admission	8058 (52.0)	2683 (71.4)	**<0.001**	1803 (53.7)	2389 (71.2)	**<0.001**
Complication	2164 (14.0)	939 (25.0)	**<0.001**	504 (15.0)	810 (24.1)	**<0.001**
ICU LOS	3 (2–6)	6 (3–11)	**<0.001**	3 (2–6)	5 (3–10)	**<0.001**
Hospital LOS	5 (3–9)	10 (7–16)	**<0.001**	5 (3–9)	10 (7–16)	**<0.001**

Bolding denotes statistical significance.

**Figure 2:**
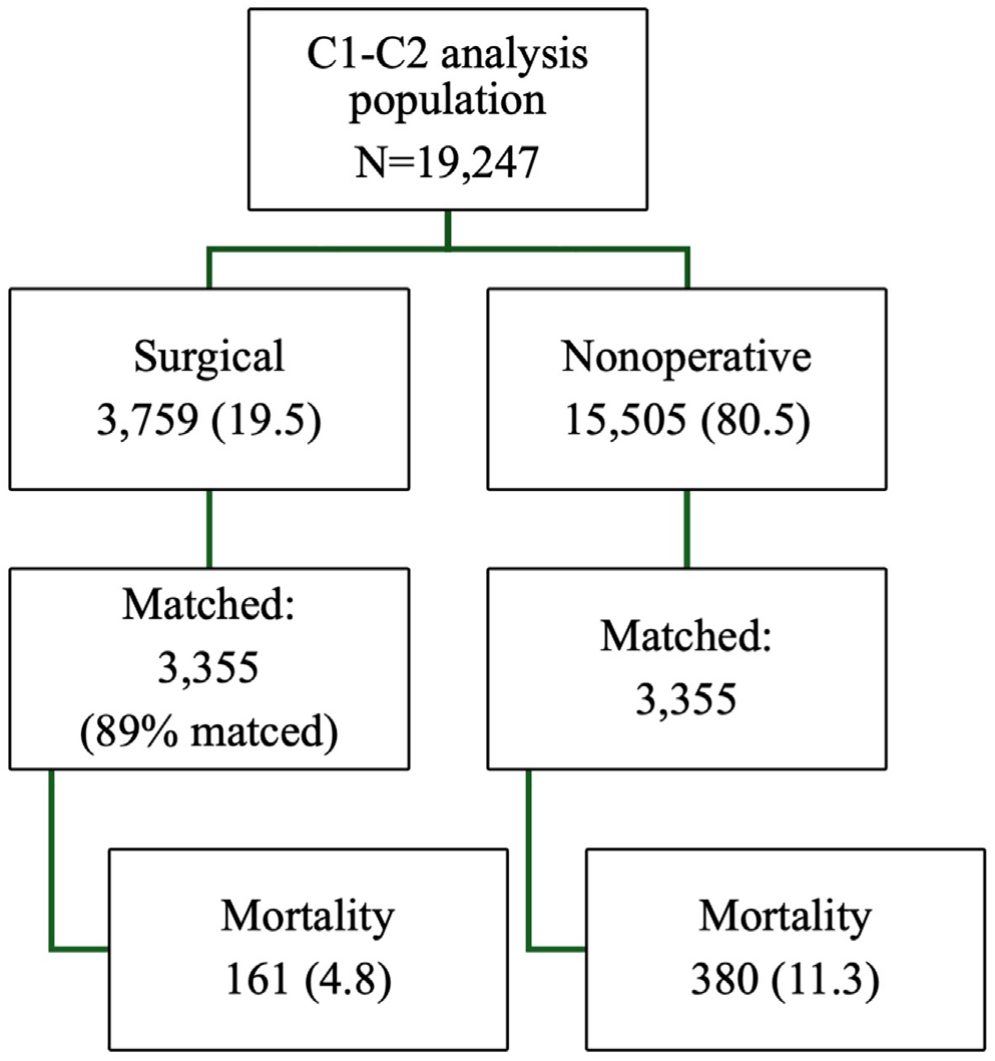
Propensity-matched analysis population.

The propensity analysis resulted in 6,710 patients: 3,355 nonoperative and 3,355 operative (representing 89% of surgically managed patients). Patients were well matched on all study covariates (Supplementary Table 1). In the matched population, mortality was significantly lower for operatively managed patients compared to the nonoperative subset (4.8% vs. 11.3%, *p* < 0.001, Figure 2). The matched patients who were operatively managed continued to have significantly worse morbidity than the matched patients who were not operated, including higher ICU admission, longer ICU LOS and hospital LOS, and a greater likelihood of developing a complication (Table 3).

When comparing outcomes in the operative group who were matched (*n* = 3,355) and unmatched (*n* = 404), there were no differences in mortality, both in-hospital and when discharge to hospice was considered, and no differences in ICU admission rates and hospital LOS (Supplementary Table 2). The matched operative subset was less likely to have a complication (24% vs. 32%) and a shorter ICU LOS (5 vs. 6 days) than the unmatched operative subset. When comparing outcomes in nonoperatively managed patients, those in the matched analysis had significantly lower mortality and worse secondary outcomes than unmatched patients (Supplementary Table 2).

## Discussion

Expert-based consensus recommendations for surgical management of combined C1–C2 fractures by the AANS/CNS are based on class III evidence only [7]. This large, population-based analysis is the first study to provide class II evidence of surgical management for patients with combined C1–C2 fractures. This study offers valuable insights into the factors influencing surgical management and their association with outcomes. We identified patient-specific and injury-specific predictors for operative intervention and used a well-matched cohort to provide evidence that surgical intervention was associated with a significant survival benefit.

This study has several limitations. First, database studies are unable to establish causality, and there is a potential bias caused by unmeasured confounders not collected in the database. Second, participation in the NTDB is voluntary and thus does not represent a true representative sample of all U.S. level I and II trauma centers. Third, the database lacks post-discharge follow-up, precluding assessment of intermediate and long-term survival and functional outcomes beyond discharge disposition. Fourth, ICD-10 codes do not capture important radiographic details such as the degree of fracture displacement, atlanto-dental interval, and union/nonunion status, which may influence surgical decision-making and prognosis. Finally, the low surgical management rate among patients ≥87 years old (7.1%) highlights potential selection bias, whereby only the healthiest patients were selected for surgery. Propensity score matching was employed to mitigate this bias, although residual confounding remains possible. Even in the matched cohort, the rate of surgical intervention in the oldest age quintile was only 8.1%, underscoring the cautious approach to operative care in this demographic.

Injury characteristics suggestive of mechanical instability – such as displaced C1 and C2 fractures, spinal cord injury, and vertebral ligament dislocation – were the strongest predictors for surgical intervention. Two large systematic reviews on the management of combined C1–C2 fractures recommended treatment based on specific characteristics of the axis fracture, namely unstable C1-type II odontoid fractures and unstable C1-Hangman fractures, or axis fracture with a disrupted transverse ligament [6, 7]. Odontoid type II fractures were the most prevalent fracture pattern in our cohort (48%), followed by posterior arch fractures (29%). Our rate of odontoid type II fractures is lower than previously reported but the rate of posterior arch fractures is consistent with prior reports. Mohile et al. noted odontoid type II fractures in 66% and posterior arch fractures in 32% of patients in their systematic review [6]. Similarly, Di Domenico et al. reported 73% of patients with odontoid type II fractures and a 30% incidence of posterior arch fractures [18]. Such differences may reflect variations in study population, institutional practices, surgeon preferences, and patient selection criteria.

Conversely, increasing age, higher frailty scores, hemodynamic instability, and GCS ≤8 were associated with a lower likelihood of surgery. The C1–C2 injury pattern predominantly affects older adults injured from falls; in our study, the median age was 76 years, and 69% were injured from falls. These findings align with Di Domenico et al., who reported a mean age of 76 years and falls as the predominant mechanism of injury (85%) [18]. Age was an independent predictor of surgical management, where increasing age quintile was associated with decreased likelihood of surgical intervention. Likewise, frailty was also independently associated with decreased odds of surgery. In a previous investigation, Salottolo et al. found that age was the primary determinant for surgery in odontoid type II fractures, supporting the present analysis [19]. Age and frailty likely play critical roles in determining candidacy for surgery in this vulnerable population [13]. These patient- and injury-specific characteristics were not previously identified as factors for surgical management in prior large systematic reviews [6, 7].

Importantly, surgical intervention was associated with significantly lower mortality. This survival benefit persisted after generating a representative, propensity-matched cohort, further supporting the benefit of surgical management in appropriately selected patients. Our results are consistent with a study by Foote et al. that demonstrated a survival benefit associated with surgical management in patients with odontoid type II fractures using a propensity-matched NTDB cohort [20]. Despite the observed survival benefit, surgical intervention was associated with worse secondary outcomes, including higher rates of ICU admission, increased complications, and longer hospital length of stay.

## Conclusions

Fracture characteristics suggestive of mechanical instability were associated with surgical management, whereas age ≥70 years, frailty, hemodynamic instability, and GCS ≤ 8 were associated with a lower likelihood of surgery, highlighting the balance between fracture patterns and patient resilience. Surgical intervention for combined C1–C2 fractures was associated with significantly improved survival compared to nonoperative management, even after propensity score matching. This survival advantage must be weighed against the increased risk of complications, greater ICU utilization, and longer hospital stay. Future prospective studies should clarify the relative benefits of specific operative techniques (e.g., occipitocervical fusion, posterior fusion, anterior fixation) and nonoperative approaches (e.g., cervical collar versus halo immobilization) to optimize outcomes in this high-risk population.

## Data Availability

The datasets used and/or analyzed during the current study are publicly available. The NTDB remains the full and exclusive copyrighted property of the American College of Surgeons. The American College of Surgeons is not responsible for any claims arising from works based on the original Data, Text, Tables, or Figures.

## Supplementary material

*Supplementary Table 1*: Differences in characteristics in the propensity matched population. *Supplementary Table 2*: Outcomes of matched compared to unmatched patients. The supplementary material of this article is available at https://sicotj.edpsciences.org/10.1051/sicotj/2025058/olm.
