# Supplementary material for: Predictors of surgical management and its impact on outcomes for combined C1–C2 fractures: National registry study
Source: SICOT J. 2026 Jan 6;11:59. doi: 10.1051/sicotj/2025058 (PMC12779261; doi:10.1051/sicotj/2025058)
Supplement: Supplementary file 1 — Supplementary Table 1: Differences in characteristics in the propensity matched population. Supplementary Table 2: Outcomes of matched compared to unmatched patients. [file sicotj-11-59-s1.pdf]

Supplementary Table 1. Differences in characteristics in the propensity matched population

Characteristic	Operative (matched, 3,355)	Nonoperative (matched, 3,355)	P value
Age quintile			0.31
< 55	719 (21.4)	667 (19.9)	
55-69	878 (26.2)	853 (25.4)	
70-79	915 (27.3)	967 (28.8)	
80-86	561 (16.7)	594 (17.7)	
≥87	282 (8.4)	274 (8.2)	
Male sex	1983 (59.1)	2002 (59.7)	0.64
White race	2855 (85.1)	2899 (86.4)	0.12
Frail (≥2m MFI-5)	1084 (32.3)	1085 (32.3)	0.98
ED GCS ≤8	279 (8.3)	258 (7.7)	0.34
ISS ≥25	374 (11.2)	329 (9.8)	0.07
ED hemodynamic instability	209 (6.2)	179 (5.3)	0.12
Concomitant injuries			
Cervical ligament sprain	532 (15.9)	509 (15.2)	0.44
Vertebral dislocation	332 (9.9)	300 (8.9)	0.18
Traumatic brain injury (TBI)	567 (16.9)	542 (16.2)	0.41
Spinal cord injury (SCI)	566 (16.8)	528 (15.7)	0.21
Specific C1 and C2 fractures			
Odontoid type II fracture	1780 (53.1)	1841 (54.9)	0.14
Odontoid type I/III fracture	552 (16.5)	504 (15.0)	0.11
Jefferson burst fracture	340 (10.1)	346 (10.3)	0.81
Posterior arch fracture	974 (29.0)	1009 (30.1)	0.35
Lateral mass fracture	389 (11.6)	367 (10.9)	0.40
Other C1 fracture	1049 (31.3)	1028 (30.6)	0.58
Displaced C2 fracture	2955 (88.1)	2987 (89.0)	0.22
Displaced C1 fracture	2450 (73.0)	2441 (72.8)	0.80

Supplementary table 2. Outcomes of matched compared to unmatched patients

Outcome, n (%) or median (IQR)	Operative			Nonoperative		
	Unmatched (n=404)	Matched (n=3,355)	p value	Unmatched (n=12,150)	Matched (n=3,355)	p value
Mortality	28 (6.9)	161 (4.8)	0.06	1969 (16.2)	380 (11.3)	**<0.001**
Morgue/hospice	32 (7.9)	214 (6.4)	0.24	2469 (20.3)	481 (14.3)	**<0.001**
ICU admission	294 (72.8)	2389 (71.2)	0.51	6255 (51.5)	1803 (53.7)	**0.02**
Complication	129 (31.9)	810 (24.1)	**<0.001**	1660 (13.7)	504 (15.0)	**0.04**
ICU LOS	6 (3-12)	5 (3-10)	**0.02**	3 (2-6)	3 (2-6)	**0.01**
Hospital LOS	11 (7-17)	10 (7-16)	0.15	5 (3-8)	5 (3-9)	**0.001**
